# Use of Gaussian process regression for radiation mapping of a nuclear reactor with a mobile robot

**DOI:** 10.1038/s41598-021-93474-4

**Published:** 2021-07-07

**Authors:** Andrew West, Ioannis Tsitsimpelis, Mauro Licata, Anz̆e Jazbec, Luka Snoj, Malcolm J. Joyce, Barry Lennox

**Affiliations:** 1 Department of Electrical and Electronic Engineering, University of Manchester, Manchester, M13 9PL UK; 2 Department of Engineering, Lancaster University, Lancaster, LA1 4YW UK; 3 Joz̆ef Stefan Institute, Jamova cesta 39, 1000 Ljubljana, Slovenia

**Keywords:** Electrical and electronic engineering, Nuclear energy

## Abstract

Collection and interpolation of radiation observations is of vital importance to support routine operations in the nuclear sector globally, as well as for completing surveys during crisis response. To reduce exposure to ionizing radiation that human workers can be subjected to during such surveys, there is a strong desire to utilise robotic systems. Previous approaches to interpolate measurements taken from nuclear facilities to reconstruct radiological maps of an environment cannot be applied accurately to data collected from a robotic survey as they are unable to cope well with irregularly spaced, noisy, low count data. In this work, a novel approach to interpolating radiation measurements collected from a robot is proposed that overcomes the problems associated with sparse and noisy measurements. The proposed method integrates an appropriate kernel, benchmarked against the radiation transport code MCNP6, into the Gaussian Process Regression technique. The suitability of the proposed technique is demonstrated through its application to data collected from a bespoke robotic system used to conduct a survey of the Joz̆ef Stefan Institute TRIGA Mark II nuclear reactor during steady state operation, where it is shown to successfully reconstruct gamma dosimetry estimates in the reactor hall and aid in identifying sources of ionizing radiation.

## Introduction

Radiological characterization covers a broad range of tasks undertaken at nuclear facilities, be it for general operations and decommissioning^1^, safeguarding and security or emergency response^2,3^. An important output from any characterization study is an estimate of dose rate within a facility, which can be used to support planning and execution of activities to minimise the risks to health associated with exposure to ionizing radiation^4^. Furthermore, if available on a regular basis then dose rate measurements can also be used as an indicator of changes in operational conditions. For example, an increase or decrease in dose rate at a specific location may indicate a fault or incident related to unexpected material release or a change in activity. Therefore, dose rate can act as a proxy for asset health, especially when sources may be concealed or not easily visualized.

The task of collecting radiation measurements from a nuclear facility has traditionally been performed manually by human operators carrying radiation monitoring equipment or with equipment attached to their person^5,6^. However, given the need to reduce any risks associated with the manual collection of radiological measurements in environments, and to follow the principles of ALARP (as low as reasonably practicable)^7^, there is a strong desire to deploy remotely operated robots to collect radiological measurements *in-situ*. Robots are expendable, more tolerant to radiological, chemical and biological hazards, and can operate for longer periods of time in radiation environments compared to humans. Furthermore, elevated risks associated with some nuclear facilities can preclude human entry entirely, leaving remote vehicle deployment as one of the few viable solutions for inspection tasks. As a result of the clear benefits conferred by *in-situ* sampling, the use of robotic systems has seen an increase in the nuclear sector, in part due to the response to the on-going situation at the Fukushima–Daiichi nuclear site and surrounding area. Recognising the potential that robotic systems have, the Nuclear Decommissioning Authority in the UK have stated that one of its four “Grand Challenges” is to reduce the activities carried out by humans in hazardous environments by 50% before 2030^8^. There is a strong desire to use Unmanned Ground Vehicles (UGVs) to conduct routine radiological surveys, with such systems already being used extensively in the extreme environments of Fukushima–Daiichi and Chernobyl^7,9,10^.

The robotic collection and subsequent analysis of radiation measurements is beginning to have direct consequences to human and robot health. For example, the Quince robot deployed at Fukushima was used to undertake preliminary surveys to better prepare for future inspection of the primary containment vessel and for plant workers to enter Unit 3 to restore the core spray system^11^. Stakeholders require clear and informative data for effective planning and risk mitigation, however, point observation dose measurements collected during such surveys lack the descriptive power of full interpolated maps^12^. Interpolation of robot-retrieved, spatially-resolved, point observation data must be carefully processed to ensure that it provides both a visually descriptive reconstruction for broader context and a physically realistic reconstruction of nuclear environments to allow stakeholders to make informed decisions.

Although numerous UGVs have been developed for the purposes of characterizing radiation environments^7,13^, very few studies have demonstrated how radiation maps can be constructed from surveys of facilities containing actual nuclear materials^14–17^. In the work presented here, radiation data is collected from two real-world nuclear environments, namely the Lancaster University Neutron Laboratory and the Joz̆ef Stefan Institute (JSI) TRIGA Mark II nuclear reactor. The measurements collected from these facilities exhibit qualities which, when combined, cannot be catered for using existing analysis and interpolation techniques. Specifically, issues related to irregularly spaced data, non-point radiation sources, inherent fluctuation in radiation intensity due to the stochastic decay of radioactive materials, and repeat observations at the same location, need to be addressed if robotic surveys of radiation facilities are to be used to help inform decision making in the nuclear industry.

Several techniques that have been developed for generating radiation maps for outdoor use, or larger indoor spaces rely on regular spacing of the radiation measurements that are collected^18–20^. However, the reality of unknown geometries and objects in unstructured nuclear environments means that it is not always possible to collect regularly spaced measurements. Furthermore, the resolution of the interpolated map is intrinsically linked to the resolution of the sampling grid, which in two or three dimensions can quickly become time consuming and burdensome. For example, Lazna et al^15^ used a regular spacing of 1 m and delaunay triangulation for interpolation, whereas Zakaria et al^14^ presented linearly interpolated observations based on a regular grid. As well as physical restrictions, manual teleoperation, minimisation of ionizing radiation dose to electronics, and other autonomous behaviours will almost certainly necessitate a departure from regular spaced sampling during any robotic survey. Therefore, an interpolation method is required which can be applied to data that has been collected at irregular locations.

Mapping and or localisation of radiation sources using UGVs typically involves the approximation of solely point radiation sources using methods that either assume a predefined number of measurements^16,18^ or require the estimation of the number of sources in the environment^17,21^. Although the majority of radiation sources can be approximated as isotropic point-sources, as will be discussed later, there are examples of non-point source radioactivity that can be found during surveys of nuclear facilities, which will introduce errors into any interpolated radiation map, if not considered. Furthermore, in cluttered environments, effects such as attenuation and scattering of gamma photons lead to a distinct departure from an isotropic radiation field. Previous work utilising real radiation sources typically place the source in the middle of a large open space, far from other objects^14–17,19,20,22,23^, whereas conscious storage of radiation sources in an active facility is likely to be in a container at the edge of a room, on a table^18^ or against a wall, often with shielding materials placed around it. If accurate radiation maps of nuclear facilities are to be generated then any interpolation method needs to account for the collimation of radiation that will result from any objects, such as shielding that may be in place around active sources within the environment.

A further challenge with generating accurate radiation maps is that measurements made by radiation detectors will have inherent uncertainty resulting from the stochastic process of radioactive decay^24^. Assuming the decay process is stochastic with a constant average intensity, then observed events will be described by a Poisson distribution. Many interpolation techniques cannot readily account for measurement uncertainty and are said to be “exact”^25^, which will introduce additional errors in any resulting interpolated map. This effect can be readily seen in Fig. 2, where irregularly spaced, Poisson distributed, radiation observations from a point-source are interpolated using linear interpolation. Where the intensity should be decreasing smoothly radially from the origin, the interpolated results follow the fluctuations in observations, producing an inaccurate reconstruction.

Fluctuations caused by the stochastic nature of radioactive decay can be addressed in part through averaging of repeat measurements, which would require the robot to be left at each location for sufficient time to record multiple measurements. Previous literature have either implemented interpolation techniques which cannot incorporate repeat samples^14,15,20^, or have discarded repeat measurements^19^, with very few studies explicitly accounting for repeat measurements. Examples of where repeat measurements were considered is by Mascarich et al^23^ and Cortez et al^22^, who used the dwell time of the robot to allow for repeat measurements to decrease uncertainty in an effort to combat low count rates. To improve the quality of any resulting survey, the interpolation strategy must accommodate repeat observations for the same input parameters, e.g. spatial location, to better cope with the inherent variation in radiation measurements.

Considering the physical properties of the radiation measurements and their collection, the broad category of Gaussian process methods was believed to be a viable solution for generating radiation maps from sparse measurements, containing noise and non-Gaussian distributions and in this study Gaussian Process Regression (GPR)^26^ was implemented. Gaussian processes are able to describe arbitrary functions through the use of sensibly constructed covariance functions. It was anticipated, in this work, that this would allow unknown complex radiation fields to be estimated accurately. GPR also provides an additional benefit, in that as well as an estimate of radiation intensity, it provides a measure of confidence in the estimate which many alternative interpolation techniques do not offer directly. This confidence metric can be invaluable for the planning of future tasks and acting to minimise radiation exposure and other risks in line with ALARP. GPR is mathematically closely related to other kernel-based approaches, namely Kriging, more commonly associated with geostatistics. Though variations of Kriging can be used to reconstruct radiation fields^27^, GPR is of particular interest from a robotics perspective, as there is existing literature regarding how it can be leveraged to direct autonomous behaviours^28,29^, and is used extensively in machine learning^26^.

Gaussian techniques have previously been explored for reconstruction of radiation environments^30,31^, however, a number of modifications to existing techniques needed to be made for it to be suitable for the irregularly spaced, repetitive, low count rate data that was collected using a UGV in this work. The case studies described in this paper demonstrate the efficacy and additional benefits that are provided when the proposed GPR approach was applied to reconstruct radiation fields of real-world unstructured nuclear environments, using radiation measurements collected from a UGV.

## Method and materials

**Figure 1:**
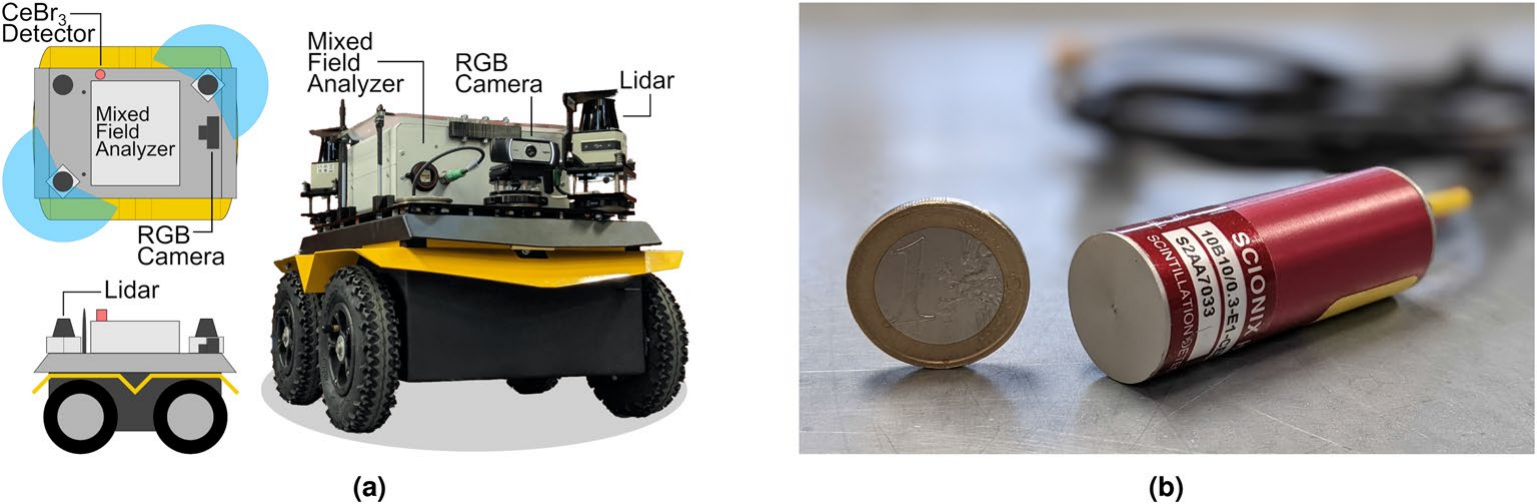
(**a**) Diagram and photograph of robot platform, consisting of a Clearpath Robotics Jackal UGV, front and rear 2D Lidar, forward facing RGB camera, and gamma radiation instrumentation (diagrams generated using Inkscape version 1.0.2 https://inkscape.org/release/inkscape-1.0.2/). (**b**) Photograph of the compact ${\text {CeBr}}_{3}$ scintillator detector used for this research.

### Robot platform

Despite the risks associated with humans entering radioactive environments, the use of robots, and in particular UGVs, in the nuclear industry remains limited and there do not yet exist recognised commercial robotic platforms designed to explore such facilities. In this study, the Clearpath Robotics Jackal UGV was selected as the robotic platform that would be used. This choice was made based on key criteria such as ground clearance for obstacle management, payload mass and space to accommodate the necessary scientific payload, whilst minimising overall size to enable access to more physically restricted locations. The Jackal is a popular and well-established platform in the robotics research community and has been adopted as a platform in previous nuclear applications^32^, which have included site deployment^33^. Whilst the Jackal platform has not been specifically designed for radioactive environments and some of its electronic systems will be susceptible to damage from radiation, the levels of radiation that were to be encountered in this study^34^, which are commensurate with many environments in nuclear power plants and decommissioning applications, were high enough to be of potential harm to humans, but not sufficiently elevated to be of risk to the electronics within the robot^35^. A diagram and photograph of the robot platform is shown in Fig. 1a. For all on-board hardware, an in-built lithium ion battery (capacity 330 Wh) provides power (5 V, 12 V, and 24 V rails) and communications is handled by a computer (via USB or ethernet), with the entire system utilising the Robot Operating System (ROS) including scientific instruments. ROS is a popular middleware which allows rapid integration of hardware and software on robotic platforms^36^, and in some cases nuclear sector robots are obligated to be ROS compatible at a governmental level^37^. The robot was capable of operating for over 2.5 h, maneuvering almost constantly, with the additional power draw and mass of the scientific payload.

As shown in Fig. 1a, to assist teleoperation of the robot, a RGB camera was placed at the front of the robot. Two SICK TiM 571 2D Lidars were mounted at opposite corners to provide overlapping $360^{\circ }$ coverage around the robot, whilst maximising the payload space in the central region of the platform. The central payload space accommodated interchangeable instrument payloads, in this work a Mixed Field Analyzer (MFA) and ${\text {CeBr}}_{3}$ scintillator detector were mounted towards the rear left side of the robot, above the MFA. To provide Simultaneous Localization and Mapping (SLAM) capabilities, the ROS package Cartographer^38^ was used in 2D mode, utilising the 2 Lidars, on-board Inertial Measurement Unit (IMU) and odometry provided by the robot platform. Communications between the remote operator and robot, through ROS, were accomplished using a consumer-grade WiFi router. Despite the additional concrete, steel and water found in nuclear environments, in this work, WiFi strength with stock dipole antennas on the router and robot were adequate in providing continued remote communications without interruption. The robot and sensor data, including radiation intensity as a coloured pointcloud, were visualized in real-time through the ROS package *rviz* (robot visualization). During remote inspection and exploration, the operator used a gaming controller (PS4) to steer the UGV, utilising the RGB camera and Lidar for navigation and situation awareness beyond visual line of sight for the majority of the mission.

### Gamma radiation monitoring

The radiation instrumentation apparatus comprised a ${\text {CeBr}}_{3}$ scintillator detector (Scionix), coupled to a quad-channel MFA from Hybrid Instruments Ltd. Such detectors are characterized by very high light output, relatively high energy resolution (between 22 and 2 % FWHM for energies ranging between 30 and 2600 keV), and low intrinsic background response^39^. For this work, the energy range of 300 to 2500 keV was used (5–2 % FWHM). Previous research has shown that pulse height spectra calibration tests for ${\text {CeBr}}_{3}$ are consistent and, when unshielded, its efficiency is stable up to an irradiation limit of 15 Gy/h^40^. Furthermore, this detector had been previously integrated on a submersible robot (AVEXIS), which was developed for Sellafield’s legacy ponds and the Fukushima–Daiichi pressure containment vessels^39,41^. As the detector used was not calibrated, count rate is employed rather than dose rate. Using approaches such as back-calibration^42^, it is possible to convert to dose rate estimates.

Figure 1b depicts the general physical dimensions of the detector in comparison to a 1 euro coin. The 10 mm diameter $\times ~10~\hbox {mm}$ length crystal was enclosed in a cylinder of 1 mm thick aluminium, 20 mm diameter and 55 mm length. Two RG174 wires from the cylinder connected a high voltage supply to the photomultiplier tube which captured the output signal of the detector. The MFA’s digitizer provided 12-bit resolution at a sampling rate of 500 MSa/s, and could be operated in Multi-Channel analyzer mode for pulse height spectroscopy, and Pulse-Shape Discrimination (PSD) mode for distinguishing between neutron and gamma events. Even though in this research it was utilised with a gamma-sensitive detector, the setup allowed flexibility to interface up to four detectors simultaneously. Furthermore, the digitizer provided dedicated TTL outputs for each channel. These yielded a pulse which corresponded to a radiation event. The gamma instrumentation was directly integrated with ROS via custom electronics^40^, to allow for automatic acquisition and fusion of time and spatial metadata from the robot SLAM implementation at a rate of 1 Hz.

### Reconstruction of complex radiation fields

GPR is capable of interpolation and also extrapolation to distances similar to the characteristic length scale of the system, in multiple dimensions. GPR can accept sparse and highly clustered data, including repeat measurements at the same location. It is also possible to include uncertainty estimates with observed data, such as Poisson variance, which is critical for the low count rates observed in this application.

**Figure 2:**
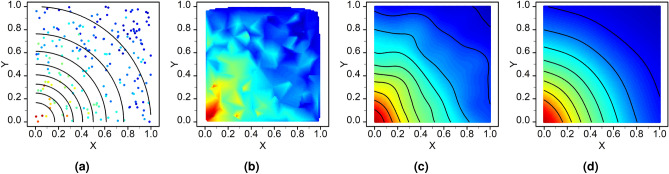
Example comparison of interpolation techniques to reconstruct the radiation field from a point source at the origin from, irregularly spaced uncertain observation data (**a**), using linear interpolation (**b**), minimum curvature thin-plate splines (**c**), and Gaussian Process Regression (**d**). (All plots generated using R version 3.6.1^43^ https://cran.r-project.org/bin/windows/base/old/3.6.1/).

The immediate efficacy of GPR and the kernel used in this work can be witnessed in Fig. 2d, where it is shown to produce smoother estimates of radiation intensity when provided with the uncertain and irregularly spaced observations shown in Fig. 2a. It produced significantly improved reconstruction of the original radiation field when compared to more commonly used methods, such as linear interpolation shown in Fig. 2b. GPR demonstrates less influence from noise when compared to minimum curvature gridding approaches (Fig. 2c) as recommended by IAEA^42^, though this approach performed considerably better than exact spline and interpolation methods, and as a kernel-based approach offers some extrapolation.

A further benefit of GPR, especially to stakeholders, is the estimate of confidence in a prediction, which ordinarily applied methods do not provide. However, as will be shown, this confidence is heavily linked to the length scale of the interpolation and where observations have been made. A significant drawback of GPR is the computation time required, scaling poorly (${\mathcal {O}}(n^{3})$) with the number of observations, which limits this technique to post-survey analysis for large datasets.

Although GPR can describe arbitrary functions, it is anticipated that many environments that will be surveyed will contain a number of point radiation sources and therefore the chosen covariance function must be capable of modelling both point source radiation as well as more complex fields when required. The Radial Basis Function (RBF) and Matérn kernels are preferable in general as they naturally return to a minimum value as distance increases. This allows for better representation of the physical nature of point-source radiation intensity, whilst still allowing the approximation of arbitrary continuous functions. Both RBF^30^ and Matérn 3/2^31^ kernels have been previously used for radiation mapping, however, they have only been applied to continuous variable dose rate with limited uncertainty. In comparative studies of RBF and Matérn kernels, that were completed in this work, it was found that a Matérn 3/2 function generally provide lower root mean square residuals when it was fitted to the inverse square radial decay of the low activity simulated point source, described by $I_{r} = I_{0} / r^{2}$, with Poisson variance. This finding is consistent with previous work on surrogate models for radiation sources^44^.

Equation (1) shows the covariance function used in this work. It consists of a Matérn 3/2 function, isotropic in euclidean space^45^, with radial distance, *d*, and with scale length *l*, in summation with a bias function *b* to mimic an offset in counts due to existing homogeneous background radiation. The background rate is likely to be specific to particular environments and therefore, explicitly including a background rate allows for greater flexibility and generalisation of scenarios.

1 $$\begin{aligned} k(d) = \left( 1 + \frac{\sqrt{3}d}{l}\right) \exp \left( -\frac{\sqrt{3}d}{l}\right) + b \end{aligned}$$

Observation uncertainty would typically be treated as being normally distributed for mathematical simplicity^20^. However, as radioactive decay is a random Poisson process, only at high count values does the uncertainty tend to a normal distribution. For the environments that were surveyed in this work, it was expected that count rates would remain at a low value, particularly when measuring background levels, and therefore this approximation would not be appropriate^27^. In this work, therefore, the likelihood function was maintained as Poissonian to better account for the physical reality of measured integer count rate, at the expense of computational resources. The regression was undertaken in log-space to ensure that all estimates had values $\ge 0$, maintaining physical accuracy, i.e. it should not be possible to estimate a negative count rate.

To compute the regression, GPy a well-established package for python was used^46^. It was assumed the radiation field was time invariant (as both the laboratory source and reactor were operated at steady state conditions), only varying over euclidean space. As the detector position in this work did not vary in *z*, the location of the sensor in only *x* and *y* dimensions was necessary. The unstructured survey data was input to the regression, with estimated radiation intensity sampled at regularly spaced intervals. Once processed, the radiation reconstruction was overlaid onto an occupancy grid map provided by SLAM.

To assess the feasibility of using GPR to accurately reconstruct radiation fields from sparse measurements collected from a remote robotic vehicle, where traversal is limited, an initial test was conducted at the Lancaster University Neutron Laboratory.

## Results

### Benchmarking

The Lancaster University Neutron Laboratory consists of a 13 MBq californium-252 ($^{252}$Cf) source encased in a 0.93 m x 0.93 m x 0.9 m cuboid container of light water, with 33 mm thick steel walls. The source was exposed to one wall of the container, producing a gamma radiation field both directly and through neutron activation of other materials. The source had a simple geometry, allowing it to be modelled using Monte Carlo particle transport simulations. The resulting modelling and experimentally generated radiation maps could then be compared to assess the feasibility of robotic radiation mapping using GPR. The laboratory room and $^{252}$Cf source were reproduced using the Monte Carlo simulation tool MCNP6^47,48^. The source was modelled as generating both neutrons and gamma rays according to the typical $^{252}$Cf spontaneous fission spectrum^49^, and with the approximation of an isotropic emission profile. Though $^{252}$Cf is primarily a neutron source, the scale of the facility and sufficient gamma ray production makes it a convenient room-scale environment for testing UGVs for gamma only mapping.

**Figure 3:**
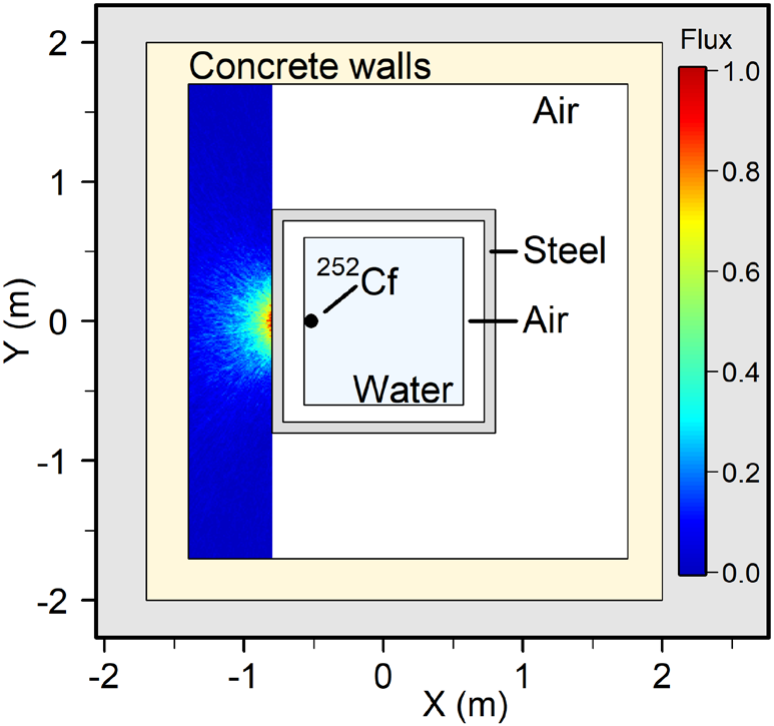
MCNP simulation of gamma ray flux at the Lancaster University Neutron Laboratory, normalised to colour scale, blue indicates low flux, red indicates maximum flux. (Generated using R version 3.6.1^43^ https://cran.r-project.org/bin/windows/base/old/3.6.1/).

Figure 3 shows results from MCNP simulation for the expected radiation field between the source containment and wall of the laboratory. The flux was evaluated by detecting the average number of gamma rays per ${\hbox {cm}}^{2}$. This was integrated between 200 keV and 2500 keV, thus covering the operational range of a ${\text {CeBr}}_{3}$ detector. Furthermore, with simulated neutrons the MCNP6 model accounts for prompt gamma production by neutron-induced reactions. Figure 3 shows field intensity reduces radially from the internal source, being attenuated before leaving the water and steel tank and propagating in air. Gamma ray flux decreased rapidly in air to a distance of approximately 0.4 m in the $-x$ direction from the tank and $\pm 0.3 \, \hbox {m}$ in the *y* axis.

For the experimental results, the robot, with integrated ${\text {CeBr}}_{3}$ detector was deployed and teleoperated from outside the laboratory. The robot entered the facility and performed two loops around the $^{252}$Cf source before returning to the origin. The time to complete the survey was less than seven minutes ($\approx 400$ data points at 1Hz publish rate). SLAM produced an occupancy grid, including obstacles which are present in the facility, but are not accounted for in MCNP calculations. As the robot traversed the environment, the radiation count data, measured by the ${\text {CeBr}}_{3}$ detector were extracted to a text file and tagged with spatial information, obtained from SLAM, that located the radiation instrumentation within the facility. Although the radiation fields characterized in this work contained significant neutron fluxes, the ${\text {CeBr}}_{3}$ detector was unresponsive to neutrons and only registered gamma photon events. As the ambient radiation field intensity increased, more gamma rays impinged on the detector, leading to an increase in photon counts registered by the ${\text {CeBr}}_{3}$ detector and reported by the MFA. In this test, the number of events (counts) over a period of one second was used to provide a relative measure of the ambient radiation field as the robot moved around the facility.

**Figure 4:**
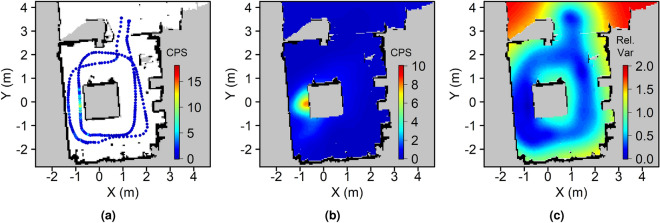
SLAM generated occupancy grid for the Lancaster University Neutron Laboratory, with spatially-resolved radiation count rate observations superimposed (**a**), and the interpolated map of radiation intensity (**b**). Blue indicates low (background) to red indicating higher counts per second. Part (**c**) shows relative estimate confidence, with blue indicating high confidence (low relative variance) and red indicating low confidence. (All plots generated using R version 3.6.1^43^ https://cran.r-project.org/bin/windows/base/old/3.6.1/).

Figure 4a shows the individual radiation count data that was measured as the robot moved around the environment containing the $^{252}$Cf source. Grey cells indicate unknown cells, black indicate cells containing obstacles (impinged by Lidar), and white cells indicate free space (no obstacles occlude Lidar). The count rate increased as the robot approached the radiation source, with lower cps in blue and higher cps through green to red. The average background intensity was $\approx 0.5$ cps with a maximum recorded value of 18 cps in front of the source. Count data was processed using GPR to produce a 2D map of the radiation field overlaid onto the map shown in Fig. 4b, at the same cell centers and resolution of 5 cm. A peak in radiation intensity surrounding the $^{252}$Cf source is apparent, decreasing radially from the internal source. The radiation flux was mostly diminished at distances beyond 0.6 m from the tank, extending approximately $\pm 0.5 \, \hbox {m}$ in the *y* axis. Figure 4b can be seen to be in excellent agreement to the MCNP6 calculations in Fig. 3, capturing the overall trend of the radiation field including prediction of the source originating inside the water tank and only background levels of radiation measured at distances beyond approximately 0.5 m from the $^{252}$Cf source. The radiation map in Fig. 4b does have some small differences from that estimated using MCNP6.

These discrepancies are believed to be because the simulation does not consider ambient background radiation nor delayed gamma rays^50^, and there is inherent uncertainty in the GPR reconstruction. However, the results indicate that applying GPR to radiation measurements collected using a robotic vehicle is capable of accurately estimating the radiation field around a radioactive source. Figure 4c shows the relative confidence in the interpolation estimate, i.e. the reported variance normalised to the estimate. An important observation is that although GPR can provide an estimate of radiation intensity across the laboratory, the confidence in this estimate will be lower in regions that were relatively unexplored. Stakeholders, radiation workers, and robotic systems can use this confidence indication as a measure of trust in these estimates and modulate their behavior or response accordingly. Furthermore, this measure of confidence can be used to drive robot behaviours for future autonomous inspection, with systems which attempt to increase data quality based on heuristics derived from both estimate and confidence.

### JSI TRIGA mark II reactor

With the proposed approach to data collection and its subsequent analysis benchmarked against a characterized radiation field, the remote system was demonstrated in the unknown and complex radiation field environment around the JSI TRIGA Mark II reactor, located in Slovenia. As described in further detail later, the radiation field consisted of collimated, attenuated, and scattered radiation, with contributions from multiple extended non-point sources, and some point approximated sources. This environment acted as a realistic surrogate for unknown hazardous environments which may be found in radiological characterization challenges. Though the reactor does produce neutrons, only gamma rays were characterised in this experiment.

**Figure 5:**
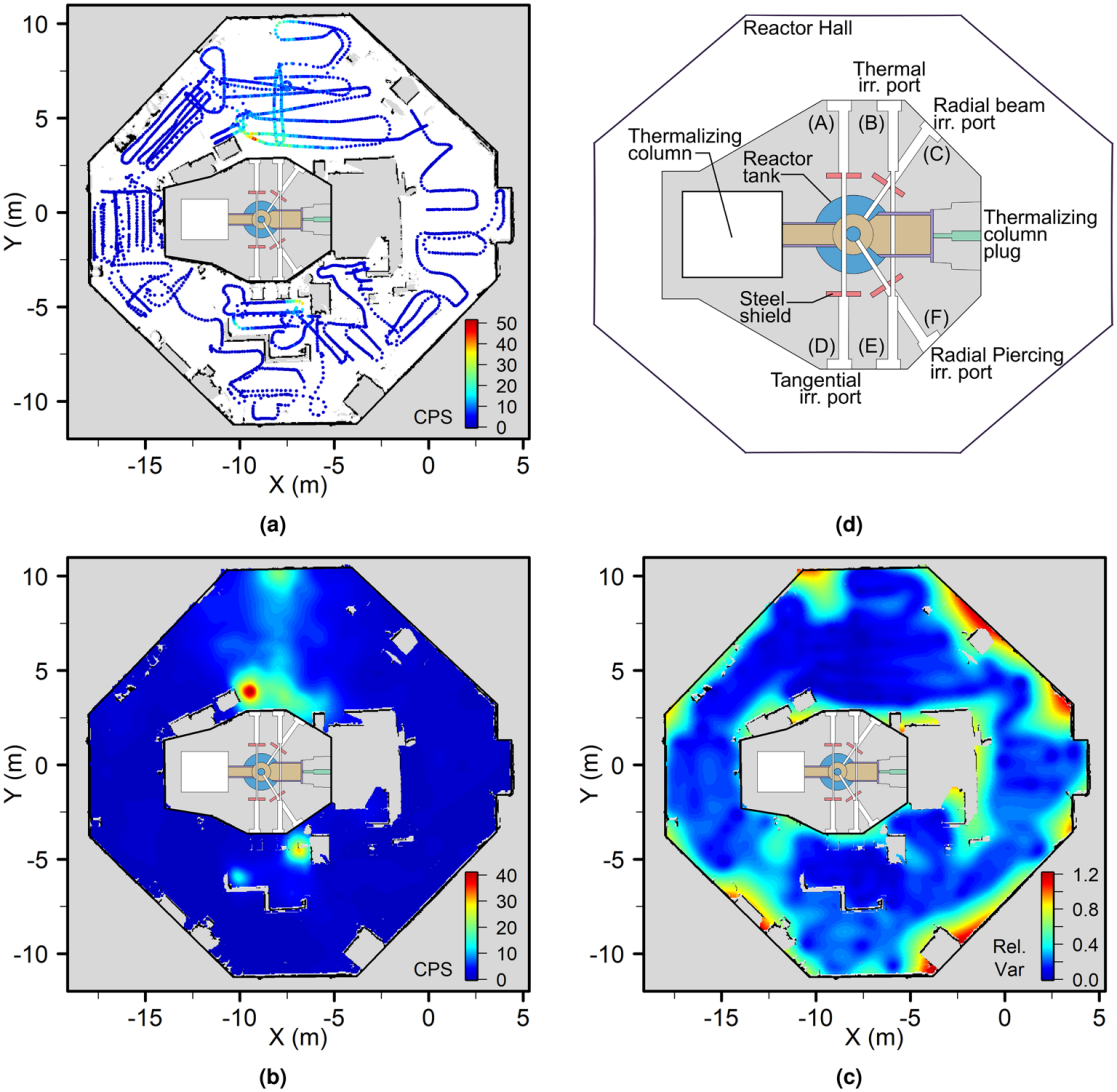
Spatially-resolved gamma radiation data overlaid on an occupancy grid generated through SLAM (**a**), and the interpolated map of radiation intensity (**b**). Blue indicates low (background) count rate through to red indicating greater counts per second. Part (**c**) shows the relative estimate variance, with blue being high confidence (low variance) and red representing low confidence. Part (**d**) shows a top view schematic of the JSI TRIGA Mark II reactor. The core is situated roughly central in the octagonal reactor hall, with irradiation ports marked by letters A–F. (Plots (**a**)–(**c**) generated using R version 3.6.1^43^ https://cran.r-project.org/bin/windows/base/old/3.6.1/, diagram (**d**) generated using Inkscape version 1.0.2 https://inkscape.org/release/inkscape-1.0.2/).

The reactor core at the JSI reactor is approximately in the centre of the reactor hall, with a staircase allowing access to a platform at the top of the reactor, with the control room located outside the reactor hall. At the base of the reactor, roughly inline with the reactor core, are a number of irradiation beam ports which can be opened for experimental purposes. These ports are identified A–F on Fig. 5d. Irradiation ports D and E, and the thermal column plug were shielded with temporary walls, with previous and ongoing experiments being operated at these locations. Thermal irradiation port B was unshielded and remained open during the survey, producing a mixed radiation field of gamma rays and thermal neutrons in the main reactor hall^51^. Further details of the facility can be found in work by Ambrožič et al^52^.

The UGV was teleoperated from the top of the reactor platform, whilst the survey was performed on the floor of the reactor hall. The reactor was operated at 1 kW during the experiment, and human access to the reactor hall floor was prohibited. Total time to complete the survey was less than 1.5 h. Spatially-resolved radiation data was collected along with a map produced by the SLAM implementation on the robot. As shown in Fig. 5a the robot completed a loop of the reactor hall whilst being manually piloted to sweep back and forth in sections, concentrating on close proximity to objects and walls where more activity was anticipated in this scenario. No attempt was made to optimise the route of the robot during teleoperation, but it is anticipated that future automated exploration of environments such as this may dramatically decrease survey time. The radiation count rate increased in front of the open beam port B ($x=-7.5~\text {m}$, $y=2.5~\text {m}$), as well as in the shielded area close to ports D and E ($x=-8.0~\text {m}$, $y=-5.0~\text {m}$).

**Figure 6:**
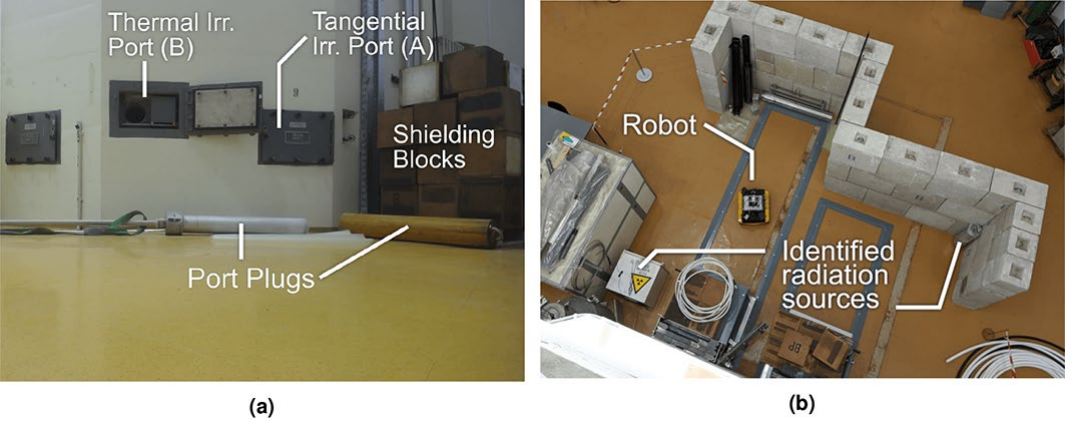
Photographs highlighting radiation sources in human restricted areas, (**a**) collected by the robot near an open irradiation port, and (**b**) discovered unknown sources (radionuclide storage container and irradiated heavy water canister). (Annotations included using Inkscape version 1.0.2 https://inkscape.org/release/inkscape-1.0.2/).

The resulting radiation map obtained by applying GPR to the radiation measurements collected is shown in Fig. 5b. The reconstructed radiation map is at a 0.05m resolution, equal to the SLAM occupancy grid, with each estimate centred at each cell marked as free space. This figure shows an increase in radiation intensity close to the beam ports A-C and a region extending in the positive *y* direction from port B towards the wall of the reactor hall where another increase in count rate is observed. Due to the long aspect ratio of the beam port the exiting gamma radiation is highly collimated and with the radiation detector on the UGV below the height of the beam port, only a partial increase in the radiation field due to scattering was observed. The height of port B is $\approx 1.12~\text {m}$, with the ${\text {CeBr}}_{3}$ detector at a height of $\approx 0.38~\text {m}$ when mounted on the robot. Despite this, the collimated radiation beam was correctly identified in the reconstruction as originating from irradiation port B. Future work will investigate the effect of adjusting the height of the radiation detector during the survey. At the wall opposite port B, partial backscattering of gamma radiation back into the reactor hall^34^ increased the measured local count rate at the lower height of the detector. This resulted in the higher radiation field recorded in this area. The hot spot adjacent to beam port B highlighted the possible influence of a neutron source that was housed in the reactor hall behind paraffin shielding blocks (depicted as a missing square directly adjacent in the reconstruction). However, the main source of the increased radiation measurements in this region was the irradiated port plugs, located on the floor in this vicinity. Figure 6a shows an image collected by the UGV, highlighting the shielding blocks in front of the neutron source, the beam port and two irradiated plugs.

At ($x=-8.0~\text {m}$, $y=-5.0~\text {m}$) two regions of elevated radiation intensity were visible within the walled off region close to the reactor, an area where human access was strictly prohibited when the reactor was in operation as beam ports D and E were open but shielded by equipment within the exclusion area. Figure 6b shows a photograph taken from the reactor platform whilst the UGV was in this restricted space. For remote decommissioning activities, it will be necessary for robotic inspections to be performed in locations where human entry is restricted. The robotic system in this experiment is small enough to bypass physical restrictions, making it capable of surveying otherwise unknown locations. The sources of radiation hot spots can be identified in Fig. 6b: a radiation source container clearly labelled with ISO ionizing radiation warnings in the bottom left corner and a tall aluminium canister located in the upper right of the photo, which is a weaker source of radiation. Upon further inspection, this canister was revealed to be a heavy water tank which had become slightly activated after being previously installed in an irradiation beam port to moderate neutrons^53^. Neither of these sources were disclosed to the UGV operators prior to or during the survey, further demonstrating how remote inspection can reveal otherwise unknown radiation sources.

## Discussion

Users should interpret the results from interpolation carefully, and try to corroborate with other information, such as SLAM maps, 3D reconstructions, and images as has been demonstrated in this work. The kernel may preferentially generate point source type features over distributed features, therefore introducing some artifacts into the reconstruction. The hot spot near the port plugs (Fig. 5b) would suggest a stronger point source or object, however, the complex interactions in this area means it is not possible to know if this is an artifact or a true approximation of the many gamma ray contributions. As observations were made very close to this region, it can be anticipated that the reconstruction holds validity in identifying a localised area of increased gamma count rate despite the overall shape, supported by the existence of likely gamma sources from images. The maps generated still provide information regarding an unknown environment and should be used as a tool to support future activities or further survey missions. Coverage which attempts to bound the available free space, then make subsequent observations internal to a bounding perimeter is likely an effective approach to improve the reconstruction and improve confidence.

As mentioned previously, the indication of confidence generated by GPR in Fig. 5c is closely related to the proximity and quality of other nearby observations. When comparing Fig. 5c to where observations were made in Fig. 5a, such as at ($x=-12.5~\text {m}$, $y=-6.0~\text {m}$), measurements collected in a single line with no complimentary information from adjacent observations is undesirable. Though observations do not necessarily need to be equally dense in both orthogonal directions, e.g. such as at ($x=2.0~\text {m}$, $y=0.0~\text {m}$), more observations in both dimensions yield better confidence in the estimated dose rate. Previous work raised concerns that vehicle-based sampling cannot provide the radiation observation density required for GPR^17^. As the robot is travelling at a reasonably slow speed, sparsity of observations in the axis of travel is not an issue, however, estimates in the region surrounding these observations which lack supporting orthogonal observations should be interpreted critically. Previous use of robot derived data and Gaussian Processes have demonstrated that high density observations are not a necessity for useful estimations^54^.

By using an appropriately chosen kernel, the reconstruction in Fig. 5b demonstrates how a robotic system can access a radioactive environment and through application of GPR, provide an accurate estimate of complex radiation fields. The use of GPR and more specifically, the use of a Poisson approximation of likelihood in this work was motivated by the low count rates observed in the relatively low activity environments examined. During the survey of the JSI reactor hall in Fig. 5a, the highest individual measurement was 51 cps, with an average background activity of $\approx 1.5~\hbox {cps}$. In the first case study, a maximum cps of only 18 was recorded directly in front of the source, yet the reconstruction still performed well in providing a meaningful representation of the radiation field. With such low cps measurements, as well as relatively little spatial resolution in front of the source, this demonstrates how powerful and robust the analysis can be. Low observed count rates mean that Gaussian approximations for uncertainty are for the most part unsubstantiated, and a Poisson treatment is more correct in this case. For certain detectors and environments with greater radiation intensity, higher count rates in hundreds or thousands per second^55^ may be amenable to a more traditional treatment of uncertainty, with a resulting reduction in computational overhead.

For decision makers, the added value afforded by UGV inspection is significant. Use of a UGV in the presence of an operating reactor core, together with the proposed GPR technique for interpolating radiation measurements provides strong evidence for the future use of robotic inspection as a routine activity in the nuclear sector. As a result of the work described in this paper, UGVs can begin to take a more prominent role alongside complimentary systems to provide improved understanding of radioactive environments and, as a consequence, reduce the risks to humans working in the nuclear industry.

## Data Availability

Datasets generated during this study and code used in analysis are available from Figshare repository, DOI: 10.48420/c.5323571.
